# Incidence of Episiotomy in Kasr Alainy OBGYN Hospital in Cairo, Egypt: A Cross-Sectional Study

**DOI:** 10.1155/ogi/4044738

**Published:** 2025-02-05

**Authors:** Omar Sadek, Nora Fahim, Hana Yehia, Mariam Elmashad, Farah Alaa, Abdulrahman Rakha, Ahmad Khaled, Nadine Sherif

**Affiliations:** Department of Obstetrics and Gynecology, Kasr Alainy Faculty of Medicine, Cairo University, Cairo, Egypt

**Keywords:** Africa, Egypt, episiotomy, vaginal delivery

## Abstract

**Objective:** This study aims to determine the incidence of episiotomy in Kasr Alainy OBGYN Hospital in Cairo, Egypt. The objectives include identifying and examining the reasons behind the notably higher episiotomy rates in this region, and assessing the relevance of general WHO recommendations in lower-middle-income countries.

**Design:** This observational cross-sectional study was conducted between March 1, 2022 and June 30, 2022, to determine the incidence of episiotomy among vaginal deliveries in the hospital.

**Setting:** Data was collected from patient charts at Kasr Alainy OBGYN Hospital in Cairo, Egypt.

**Patient Sample:** The total number of patient charts inspected was 1731, of which 1545 met the inclusion criteria.

**Methods:** Data were manually collected from patient hospital records at the end of each day, and entered into a standardized data-collection form. The data collected was then statistically analyzed using SPSS.

**Main Outcome Measures:** The overall incidence of episiotomy was 64%.

**Results:** When analyzed by gravidity, the incidence was found to be 97% in primigravida patients and 52% in multigravida patients. Additionally, several other associated factors were examined.

**Conclusions:** The incidence of episiotomy at Kasr Alainy OBGYN Hospital surpasses the rate recommended by the WHO. Our observations suggest that the primary contributing factors to this elevated incidence include perineal rigidity, the duration of labor, and local practice standards. Further research is recommended to explore the impact of perineal massage before and during delivery, as well as patients' lifestyle factors, on the necessity for episiotomy.

## 1. Introduction

Episiotomy, a surgical incision in the perineum during the second stage of labor [1], was first described in 1742 to prevent maternal injury and improve neonatal outcomes [2]. However, recent evidence does not support its routine use, citing risks such as infection, hemorrhage, anal sphincter damage, and delayed resumption of sexual activity [3]. A 2004 Munich study found that avoiding episiotomy reduces postpartum perineal pain without adverse effects on mother or fetus [4]. Consequently, guidelines now recommend restrictive use, with the WHO advocating for a 10% rate in normal deliveries [3]. Episiotomy is generally reserved for atypical fetal presentations, instrumental deliveries, and cases of fetal distress [3].

Epidemiological studies on episiotomy in the Middle East and Africa are scarce, yet the procedure is notably performed at rates exceeding WHO recommendations in the region. This study aims to determine the incidence of episiotomy in patients undergoing a vaginal delivery in Kasr Alainy OBGYN University Hospital between March 1st, 2022, and June 30th, 2022. The objectives include identifying and examining reasons behind the notably higher episiotomy rates in this region, and assessing the relevance of general WHO recommendations in lower-middle-income countries.

The study was conducted at Kasr Alainy University Hospital, the largest hospital in Egypt, with 5500 beds and serving over two million patients annually [5]. Widely regarded as a leading healthcare provider and medical education center in the Middle East and Africa, the hospital's practice standards offer a reliable representation of healthcare trends in the broader region.

## 2. Methods

### 2.1. Study Design

This cross-sectional study was conducted in Kasr Alainy OBGYN Hospital in Cairo, Egypt, between March 1, 2022, and June 30, 2022, to determine the incidence of episiotomy. Verbal informed consent was obtained from patients for anonymized data collection after the nursing staff explained the study's aim and nature upon admission. Anonymized data was manually collected by our data collection team from hospital records for all admitted patients in the vaginal delivery ward at the end of each day during the duration of the study. No unique patient identifiers were collected. This work adhered to the RECORD (REporting of studies Conducted using Observational Routinely-collected Data) statement [6].

This study was not registered in an international clinical trials repository, as it was an observational cross-sectional study focused on the incidence of episiotomy and associated factors. According to guidelines, study registration is typically required for interventional clinical trials, but it is not a mandatory requirement for observational studies of this nature. However, the authors acknowledge the importance of transparency and have adhered to ethical research standards throughout the study.

Data from hospital records was entered into a standardized data-collection form for analysis. The following information was gathered:

• Date of data collection
• Whether an episiotomy was performed
• Maternal age
• Gravidity
• Parity
• Gestational age at time of delivery
• Cervical diameter upon admission
• Fetal presentation
• Gestational weight
• Years of marriage

Hospital protocol generally states that all interventions should only be performed if indicated according to local guidelines, but does not specifically address episiotomy.

### 2.2. Study Sample

There were a total of 1731 vaginal deliveries during the 4-month period of the study. All patients who delivered vaginally after 24 weeks gestation were included in the study. Patients who delivered before completing 24 intrauterine weeks, which by hospital protocol is considered an abortion, and those whose episiotomy status was undocumented in hospital records were excluded.

Based on the exclusion criteria, 186 patients were excluded from the study. A total of 1545 patients were therefore included in the study, of which 1134 were multigravida.

A power analysis was conducted to determine the appropriate sample size of multigravid patients required for detecting meaningful relationships between key factors (Table 1) and the incidence of episiotomy [7]. The G∗Power software was used to calculate that a sample size of 962 is needed to achieve 90% power at a significance level of 0.05, assuming a small effect size (Cohen's *f*^2^ = 0.02).

### 2.3. Statistical Analysis

The incidence of episiotomy was determined using the data collected and organized in the standardized form. Further statistical analysis was conducted using the Statistical Package for Social Sciences (SPSS) 28 package. Descriptive statistics, chi-squared test of hypothesis independence, and ANOVA test were carried out for numerical variables. Categorical variables were defined in terms of the number, percentage, and risk estimate. A *p* value of < 0.05 was considered statistically significant.

## 3. Results

**Figure 1:**
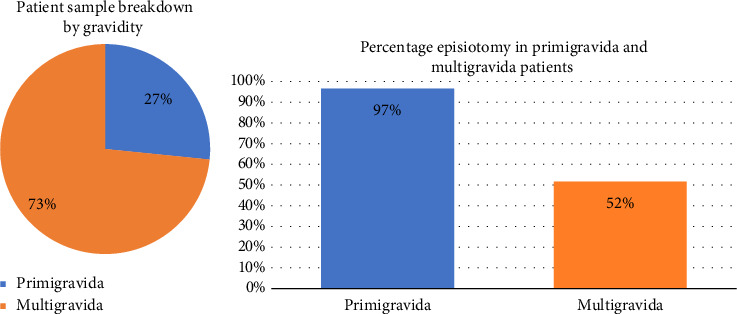
Patient sample breakdown by gravidity (a), and percentage episiotomy in primigravida and multigravida patients (b).

Of all included vaginal delivery patients, 64% had an episiotomy. Patients were further broken down according to gravidity, and it was found that of the 1545 patients, 1134 were multigravida, while 411 were primigravida (Figure 1(a)).

The rate of episiotomy was found to be 97% in primigravida patients, and 52% in multigravida patients (Table 2, Figure 1(b)). This is significantly higher than the rate recommended by the WHO (10%). Possible reasons will be discussed in the ‘Discussion' section of the paper.

Chi-squared and *p* values were calculated for all variables mentioned in the study design for multigravida patients (Table 1), primigravida patients [Table S1], and the collective patient sample [Table S2]. Tables S1 and S2 are in the “Supporting Information” file.

Statistical significance is determined by a *p* value less than 0.05. Because the incidence of episiotomy in primigravida patients was 97%, meaning that only 14 patients of the 411 did not receive an episiotomy, it was determined that findings among primigravida patients would not adequately represent clinical practices and would not be statistically significant. Findings among the collective patient sample would be highly skewed by primigravida patients and would therefore not be studied either.

Multiple variables were found to be statistically significant among multigravida; including maternal age, gravidity, parity, cervical diameter on admission, fetal presentation, and years of marriage. The correlations with these variables are discussed below, while possible reasons and deductions are discussed in the ‘Discussion' section of the paper.

1. *Maternal age* was found to be inversely proportional to the rate of episiotomy, with patients younger than 26 years exhibiting a higher-than-average episiotomy rate. This trend was particularly pronounced among patients aged 18 to 20, who had an episiotomy rate of 70%.
2. *Gravidity and parity* demonstrated a strong inverse correlation with episiotomy incidence; patients with more pregnancies and deliveries were significantly less likely to undergo the procedure.
3. *Cervical diameter* upon admission to the delivery ward was similarly inversely related to the likelihood of episiotomy. The highest episiotomy rates were observed in patients presenting with a cervical dilation of 3 cm, while the lowest rates occurred in those with cervical dilation of 7 cm or more.
4. *Fetal presentation* also strongly influenced episiotomy incidence, with all patients delivering fetuses in a noncephalic presentation requiring episiotomy.
5. *The duration of marriage* was negatively correlated with episiotomy incidence; patients married for longer periods were less likely to undergo the procedure.
  Although gestational weight and gestational age were not found to be statistically significant overall, notable trends were observed.
6. *Gestational weight* was directly proportional to the episiotomy rate, which reached 70% in newborns weighing 4 kg or more.
7. *Gestational age* was associated with an increased incidence of episiotomy in both preterm and post-term deliveries, often due to medical indications for the procedure. Among preterm deliveries, the overall incidence of episiotomy was 67%. Early preterm deliveries (before 34 weeks of gestation) had a higher incidence rate of 73% compared to 65% for late preterm deliveries (34–37 weeks of gestation).

## 4. Discussion

### 4.1. Outlook

A systematic review conducted in 2016 concluded that selective episiotomy significantly reduces the risk of severe perineal lacerations compared to both routine episiotomy and the complete avoidance of episiotomy. Additionally, episiotomies performed with angles over 40° and earlier in the second stage of labor, before crowning, seem to be more protective [8].

**Figure 2:**
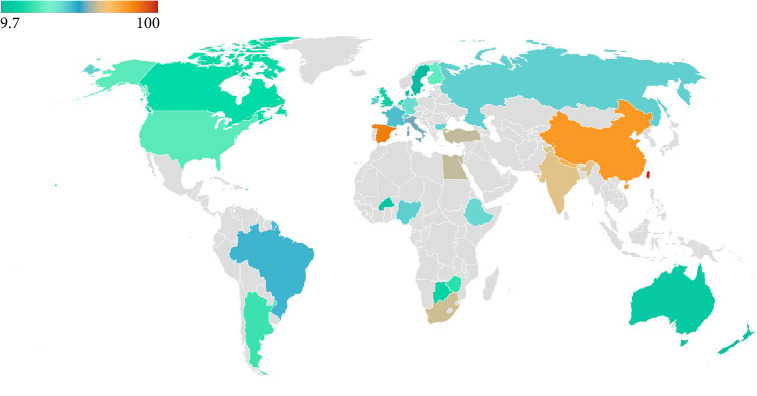
Rates of episiotomy in different countries [9, 12–16].

Episiotomy rates vary globally (Figure 2), ranging from 9.7% in Sweden to 100% in Taiwan [9]. Some countries have succeeded in dropping their episiotomy rates in the early 21st century, for instance, the United States managed to reduce episiotomy rates from 60.9% in 1979 to 24.5% in 2004 [3]. In 2003, the incidence of episiotomy was 13% in England, and 16.3% in Scotland [9]. It appears that episiotomy rates are particularly high in the Middle East, the region of our study. Oman has an episiotomy rate of 66% [15], while in Lebanon, the rate was found to be 73.3% in 2014 [16]. In Nigeria the episiotomy rate was 46.6% in 1998 [10].

### 4.2. Main Findings

The overall episiotomy rate at Kasr Alainy was found to be 64%, significantly exceeding the WHO's recommended rate of 10%. Among primigravida patients, the incidence was particularly high at 97%. Further research is needed to identify the underlying causes of this elevated rate within this patient group. Potential contributing factors can be categorized into patient-related and practice-related aspects.

Patient-related factors may include perineal rigidity, prolonged second stage of labor, and clinical indications such as instrumental deliveries, fetal distress, history or risk of obstetric anal sphincter injuries (OASIS), macrosomia, and atypical fetal presentations [17]. Practice-related factors may involve systemic issues such as limited time due to high hospital capacity utilization, understaffing, heavy patient flow, generational teaching methods, or outdated hospital protocols. A study from China similarly found that the practice of episiotomy was often influenced by practitioners' previous training, experience, and local norms rather than adherence to the latest medical evidence [18].

Among multigravida patients, the episiotomy rate was 52%. While this is notably lower than the rate among primigravida patients, it still far exceeds the WHO's recommended rate. The factors examined in the multigravida group were found to be statistically significant and clinically relevant, indicating that specific variables influence decision-making regarding episiotomy in this group. Possible explanations for the lower incidence among multigravida patients include reduced likelihood of perineal rigidity, shorter labor durations, and differences in local norms or protocols compared to those applied to primigravida patients.

### 4.3. Interpretation

Based on our observations, we concluded that the primary contributors to the increased incidence of episiotomy at Kasr Alainy OBGYN Hospital include perineal rigidity, labor duration, and local practice standards.

The effect of maternal age on the episiotomy rate is likely linked to perineal rigidity, as younger patients often exhibit a more rigid perineum. Increased perineal rigidity elevates the risk of natural perineal tears, which can be mitigated through the use of episiotomy. Additionally, unhealthy diets and sedentary lifestyles, prevalent among women from lower socio-economic backgrounds, may further contribute to perineal rigidity. While perineal massages are not routinely practiced at the hospital, future research could evaluate their effectiveness in reducing perineal rigidity.

The impact of gravidity and parity on episiotomy rates is thought to result from several factors. These include reduced perineal rigidity with subsequent deliveries, shorter labor durations, and greater patient experience and cooperation with the healthcare team, which collectively reduce the likelihood of requiring an episiotomy.

Cervical diameter serves as an indicator of labor progression, with the diameter upon admission influencing the duration a patient spends in the delivery ward [17]. The earlier a patient presents—meaning less cervical dilation—the more likely they are to undergo an episiotomy to shorten labor duration. At the hospital, episiotomy is often performed to reduce delivery time, thereby decreasing patient exhaustion and increasing the daily patient capacity of the ward, which is crucial due to the high patient influx and relative understaffing in Egyptian hospitals.

Instrumental deliveries and vaginal births of fetuses with atypical presentations are infrequent at Kasr Alainy OBGYN Hospital, as these patients are typically scheduled for cesarean sections. However, all patients who delivered vaginally with an atypical presentation underwent episiotomy.

Gestational weight and age were not found to have a statistically significant effect on the incidence of episiotomy. However, it is believed that a larger fetal head may further distend the perineum, thereby increasing the likelihood of episiotomy. Additionally, episiotomy is often performed in preterm deliveries to help manage the progression of precipitous labor.

Little is known about the post-partum effects of episiotomy in Kasr Alainy patients as typical follow-up and perinatal care programs do not cover perineal tissue health.

### 4.4. Strengths and Limitations

The study has several strengths, including

• Conducted in Kasr Alainy University Hospital, one of the largest and leading hospitals in Egypt, Africa, and the Middle East.
• First such study in Kasr Alainy University Hospital, therefore, adding useful insight into the practice of episiotomy and allowing for further research and follow up of the incidence.
• Large patient sample, 1545 patients included in the study.
• Manual, daily inspection of patient charts, insuring accuracy of collected data.

The study also had some limitations, including

• Outdated, paper-based hospital charting and record-keeping system. This makes long-term data collection especially challenging.
• Some information is not consistently mentioned in all charts.
• Indications for performing episiotomy were entirely not documented in patient charts.

## 5. Conclusion

### 5.1. General Conclusions

This study assessed the incidence of episiotomy at Kasr Alainy OBGYN Hospital in Cairo, Egypt, over a 4-month period from March to June 2022. The overall rate of episiotomy was 64%. Notably, the study identified significant correlations between episiotomy incidence and various patient factors, particularly gravidity. Primigravida patients had an episiotomy rate of 97%, compared to 52% among multigravida patients.

Given the limited availability of such data in the region and the substantial sample size of this study, the findings provide a valuable benchmark for comparing episiotomy rates regionally and globally. Furthermore, they offer a basis for evaluating the impact of hospital practices and protocols. The results also lay the groundwork for future research to explore changes in episiotomy incidence over time, postpartum perineal tissue health, and the effectiveness of interventions such as perineal massage in reducing episiotomy rates.

### 5.2. Recommendations

To enhance our understanding of episiotomy practices in the region, we recommend the following measures:

• Incorporating perineal care and follow-up into maternal postnatal care programs.
• Conducting further research on the impact of perineal massage, both before and during delivery, on perineal rigidity and the necessity for episiotomy.
• Investigating the influence of patients' lifestyle factors, particularly dietary habits and physical fitness, on the likelihood of requiring an episiotomy.

## Figures and Tables

**Table 1:** Chi-squared, *p* values, number of valid cases for each variable in multigravida patients.

Variable	Pearson chi-square	*p* value	Valid cases	Excluded cases
Maternal age	58.774	0.000381	1127	3
Gravidity	118.988	2.1527E − 21	1130	0
Parity	197.224	1.265E − 37	1130	0
Gestational age	15.090	0.656	1100	30
Cervical diameter on admission	29.264	0.001129	977	153
Fetal presentation	34.217	0.000037	1130	0
Gestational weight	61.165	0.156	849	281
Years of marriage	48.020	0.34	302	828

**Table 2:** Total percentage of episiotomy and breakdown by gravidity.

Episiotomy	Primigravida	Multigravida	Total	Percent (%)
Yes	397	586	983	64
No	14	548	562	36
Total	411	1134	1545	100
Percent	97%	52%	100%	

## Data Availability

Spreadsheet of data is available to be provided on request.

## Supporting Information

Table S1: Chi-squared, *p* values, number of valid cases for each variable in primigravida patients.

Table S2: Chi-squared and *p* values for each variable for the collective patient sample.

## References

[1] Kalis V., Laine K., de Leeuw J., Ismail K., Tincello D. (2012). Classification of Episiotomy: towards a Standardisation of Terminology. *BJOG: An International Journal of Obstetrics and Gynaecology*.

[2] Frankman E. A., Wang L., Bunker C. H., Lowder J. L. (2009). Episiotomy in the United States: Has Anything Changed?. *American Journal of Obstetrics and Gynecology*.

[3] Kartal B., Kızılırmak A., Calpbinici P., Demir G. (2017). Retrospective Analysis of Episiotomy Prevalence. *Journal of the Turkish-German Gynecological Association*.

[4] Dannecker C., Hillemanns P., Strauss A., Hasbargen U., Hepp H., Anthuber C. (2004). Episiotomy and Perineal Tears Presumed to Be Imminent: Randomized Controlled Trial. *Acta Obstetricia et Gynecologica Scandinavica*.

[5] El Dib N. A. (2015). Kasr Al Ainy, the Story of a Palace that Became a Medical School. *Kasr Al Ainy Medical Journal*.

[6] Benchimol E. I., Smeeth L., Guttmann A. (2015). The REporting of Studies Conducted Using Observational Routinely-Collected Health Data (RECORD) Statement. *PLoS Medicine*.

[7] Faul F., Erdfelder E., Lang A.-G., Buchner A. (2007). G∗Power 3: A Flexible Statistical Power Analysis Program for the Social, Behavioral, and Biomedical Sciences. *Behavior Research Methods*.

[8] Corrêa Junior M. D., Passini Júnior R. (2016). Selective Episiotomy: Indications, Techinique, and Association With Severe Perineal Lacerations. *Revista Brasileira de Ginecologia e Obstetrícia*.

[9] Graham I. D., Carroli G., Davies C., Medves J. M. (2005). Episiotomy Rates Around the World: An Update. *Birth*.

[10] Otoide V. O., Ogbonmwan S. M., Okonofua F. E. (2000). Episiotomy in Nigeria. *International Journal of Gynecology & Obstetrics*.

[11] Goldberg J., Holtz D., Hyslop T., Tolosa J. E. (2002). Has the Use of Routine Episiotomy Decreased? Examination of Episiotomy Rates From 1983 to 2000. *Obstetrics & Gynecology*.

[12] Cesar J. A., Marmitt L. P., Mendoza-Sassi R. A. (2022). Episiotomy in Southern Brazil: Prevalence, Trend, and Associated Factors. *Revista de Saúde Pública*.

[13] Indhumathi M. S., Chandra K. S. (2015). Study of Episiotomy in Our Population. *Biomedical & Pharmacology Journal*.

[14] Gebeyehu N. A., Gelaw K. A., Adela G. A., Alemu B. W., Demisie B. W., Lake E. A. (2022). The Magnitude of Episiotomy Among Women Who Gave Birth in Ethiopia: Systematic Review and Meta-Analysis. *African Journal of Reproductive Health*.

[15] Al-Ghammari K., Al-Riyami Z., Al-Moqbali M. (2016). Predictors of Routine Episiotomy in Primigravida Women in Oman. *Applied Nursing Research*.

[16] Kaddoura R., DeJong J., Zurayk H., Kabakian T., Abbyad C., Mirza F. G. (2019). Episiotomy Practice in the Middle East: A Lebanese Teaching Tertiary Care Centre Experience. *Women and Birth*.

[17] Yang J., Bai H. (2021). Knowledge, Attitude and Experience of Episiotomy Practice Among Obstetricians and Midwives: A Cross-Sectional Study From China. *BMJ Open*.

[18] Evbuomwan O., Chowdhury Y. S. (2023). *Physiology, Cervical Dilation*.

[19] Sadek O., Fahim N., Yehia H. (2023). Incidence of Episiotomy in Kasr Alainy OBGYN Hospital in Cairo, Egypt; a Cross-Sectional Study. *Authorea*.

