# Supplementary material for: Incidence of Episiotomy in Kasr Alainy OBGYN Hospital in Cairo, Egypt: A Cross-Sectional Study
Source: Obstet Gynecol Int. 2025 Feb 5;2025:4044738. doi: 10.1155/ogi/4044738 (PMC11824849; doi:10.1155/ogi/4044738)
Supplement: Supporting Information — Additional supporting information can be found online in the Supporting Information section. [file 4044738.f1.docx]

**Incidence of Episiotomy in Kasr Alainy OBGYN Hospital in Cairo, Egypt; a cross-sectional study**

**Omar Sadek, Nora Fahim, Hana Yehia, Mariam Elmashad, Farah Alaa, Abdulrahman Rakha, Ahmad Khaled, Nadine Sherif PhD**

**(This is a supplemental information file, separate from the manuscript file).**

***Supplemental Information***

*Table S1: Chi-squared, p-values, number of valid cases for each variable in primigravid patients.*

| *Variable* | *Pearson Chi-Square* | *p-value* | *Valid Cases* | *Excluded Cases* |
| --- | --- | --- | --- | --- |
| Maternal Age | 21.186 | 0.386 | 411 | 0 |
| [Gravidity](#_Gravidity) | - | - | 411 | 0 |
| [Parity](#_Parity) | - | - | 411 | 0 |
| [Gestational Age](#_Gestational_Age) | 63.303 | 2.965E-7 | 403 | 8 |
| [Cervical Diameter on Admission](#_Cervical_Diameter) | 4.825 | 0.903 | 393 | 18 |
| [Fetal](#_Type_of_VD) Presentation | 14.049 | 0.015 | 411 | 0 |
| [Gestational Weight](#_Gestational_Weight) | 24.835 | 0.937 | 305 | 106 |
| [Years of Marriage](#_Years_of_Marriage) | 5.541 | 0.902 | 174 | 237 |

*Table S2: Chi-squared and p-values for each variable for the collective patient sample*.

| *Variable* | *Pearson Chi-Square* | *p-value* | *Valid Cases* | *Excluded Cases* |
| --- | --- | --- | --- | --- |
| [Maternal Age](#_Maternal_Age) | 207.662 | 6.6616E-29 | 1542 | 3 |
| [Gravidity](#_Gravidity) | 391.222 | 6.9431E-78 | 1541 | 4 |
| [Parity](#_Parity) | 476.790 | 5.3412E-97 | 1542 | 3 |
| [Gestational Age](#_Gestational_Age) | 25.653 | 0.140138 | 1504 | 41 |
| [Cervical Diameter on Admission](#_Cervical_Diameter) | 52.375 | 9.7225E-8 | 1371 | 174 |
| [Fetal](#_Type_of_VD) Presentation | 26.402 | 0.003235 | 1545 | 0 |
| [Gestational Weight](#_Gestational_Weight) | 70.915 | 0.230739 | 1155 | 390 |
| [Years of Marriage](#_Years_of_Marriage) | 146.452 | 5.8957E-15 | 478 | 1067 |
